# Viral inosine triphosphatase: A mysterious enzyme with typical activity, but an atypical function

**DOI:** 10.1111/mpp.13021

**Published:** 2021-01-20

**Authors:** Amy M. James, Susan E. Seal, Andy M. Bailey, Gary D. Foster

**Affiliations:** ^1^ School of Biological Sciences Life Sciences Building University of Bristol Bristol UK; ^2^ Natural Resources Institute, Chatham Maritime Gillingham UK

**Keywords:** cassava brown streak disease, Euphorbia ringspot virus, ITPase

## Abstract

Plant viruses typically have highly condensed genomes, yet the plant‐pathogenic viruses *Cassava brown streak virus*, *Ugandan cassava brown streak virus*, and *Euphorbia ringspot virus* are unusual in encoding an enzyme not yet found in any other virus, the “house‐cleaning” enzyme inosine triphosphatase. Inosine triphosphatases (ITPases) are highly conserved enzymes that occur in all kingdoms of life and perform a house‐cleaning function by hydrolysing the noncanonical nucleotide inosine triphosphate to inosine monophosphate. The ITPases encoded by cassava brown streak virus and Ugandan cassava brown streak virus have been characterized biochemically and are shown to have typical ITPase activity. However, their biological role in virus infection has yet to be elucidated. Here we review what is known of viral‐encoded ITPases and speculate on potential roles in infection with the aim of generating a greater understanding of cassava brown streak viruses, a group of the world's most devastating viruses.

## Graphical abstract

What we know and do not know about the inosine triphosphatase Ham1 found in the viruses causing cassava brown streak disease, namely cassava brown streak virus and Ugandan cassava brown streak virus.

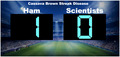

## 1 THE DISCOVERY OF VIRAL ITPASE

**FIGURE 1:**
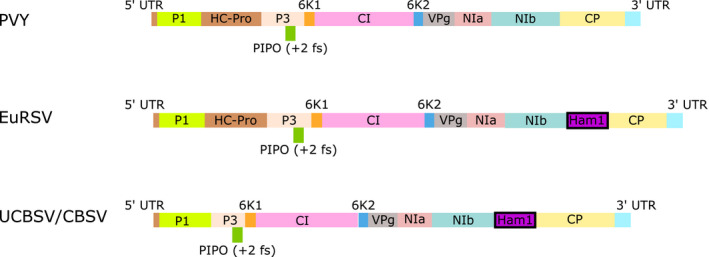
The EuRSV and (U)CBSV genomes possess a Ham1‐like gene encoding an ITPase that is absent from all other characterized viruses in the *Potyviridae* family. The type virus, *Potato virus Y* (PVY), is shown in comparison and represents the typical genome structure for this virus family. P1, the first protein, a serine protease; HC‐Pro, helper component cysteine proteinase; P3, the third protein; 6K1 and 6K2, 6‐kDa proteins; CI, cylindrical inclusion protein; VPg, viral genome‐linked protein; NIa‐Pro, nuclear inclusion body protease; NIb, nuclear inclusion body, RNA‐dependent RNA polymerase; CP, coat protein; PIPO, Pretty Interesting Potyviridae ORF, translated in the +2 reading frame

Plant viruses typically are under strong selection pressure to have a small genome size that resists the incorporation of nonessential genes. It is therefore rather surprising that a very small number of plant virus genomes, including *Cassava brown streak virus* (CBSV), *Ugandan cassava brown streak virus* (UCBSV), and *Euphorbia ringspot virus* (EuRSV), have seemingly acquired a “house‐cleaning” inosine triphosphatase (ITPase) belonging to the Ham1 family, a completely novel viral protein (Knierim et al., 2017; Mbanzibwa et al., 2009). CBSV and UCBSV are closely related single‐stranded RNA viruses belonging to the *Ipomovirus* genus, whereas EuRSV belongs to another genus in the *Potyviridae* family, *Potyvirus*. All other characterized viruses (*n* = 228; Wylie et al., 2017) belonging to the *Potyviridae* virus family, which is the largest family of RNA plant viruses, lack a gene for ITPase despite sharing an overall similar genomic structure (Figure 1).

The ITPase Ham1 in (U)CBSV and EuRSV is implicated to play an important role in the pathogenesis of these viruses because not only is it unusual for plant viruses to acquire additional genes, but in this case the same type of gene has appeared in different genera. The viral‐encoded Ham1 shows sequence motifs typical of ITPase enzymes involved in dephosphorylation of noncanonical nucleotides, atypical nucleotides that arise from oxidative damage or stresses within the purine salvage pathway.

## 2 VIRAL HAM1 IS AN ITPASE, BUT POSSIBLY NOT A SUPPRESSOR OF MUTATION

The role of viral ITPase has yet to be fully elucidated. However, expression in *Escherichia coli* and purification of the ITPases encoded by CBSV and UCBSV has shown that both are indeed functional ITPase enzymes with typical activity towards deoxyinosine triphosphate (dITP) and xanthosine triphosphate (XTP) (Tomlinson et al., 2019). The Ham1 of CBSV and UCBSV shows weak activity toward canonical nucleotides, with highest activity towards deoxyguanosine triphosphate (dGTP) and guanosine triphosphate (GTP). The relevance of the GTP/dGTP degradation is unknown; however, inosine triphosphate (ITP) may act as a competitive inhibitor for activity towards canonical nucleotide substrates in vivo.

A proposed expected function was that the viral Ham1 would help reduce mutation rates in replicating viruses. This possible role of Ham1 was evaluated in vivo by determining mutation rates of viruses infecting transgenic *Nicotiana tabacum* expressing the CBSV ITPase; however, there were no significant differences in mutation rates between wild‐type and Ham1‐expressing plants. The rates of acquisition of mutations in genomes of wild‐type CBSV compared with CBSV infectious clones (Duff‐Farrier et al., 2019) lacking Ham1 in *Nicotiana benthamiana* were also measured, but viral Ham1 did not influence mutation rates, therefore its role as a suppressor of mutation is questionable.

## 3 THE IMPORTANCE OF ITPASE IN DEPLETION OF MUTAGENIC POOLS OF NONCANONICAL NUCLEOTIDES

In all kingdoms of life, pools of noncanonical nucleotide triphosphates (NTPs) can build in the cells through normal metabolic processes or via exposure to external mutagens that result in modification of canonical nucleotides. If incorporated into DNA or RNA, noncanonical nucleotides can result in nonspecific base pairing and random mutagenesis or lead to chromosomal aberrations through error‐prone repair mechanisms. A number of different enzymes function in house‐cleaning roles to reduce the pool of noncanonical NTPs by hydrolysing pyrophosphate to give nucleotide monophosphates. There are four structurally distinct families each defined by a strictly conserved substrate specificity domain that is essential to exclude canonical nucleotides. The families are Nudix hydrolases, dUTPase, all‐α NTP pyrophosphatases (MazG), and ITPase (Maf/Ham1) (Galperin et al., 2006).

Inosine triphosphate is produced through oxidative or enzymatic deamination of purine bases or phosphorylation of IMP (Sakumi et al., 2010). Incorporation of dITP in DNA is not usually mutagenic as it is recognized, excised, and repaired, but this can lead to strand breaks and chromosomal rearrangements (Burgis et al., 2003; Waisertreiger et al., 2010). Incorporation of ITP into RNA has been shown to inhibit translation and could potentially lead to mistranslation or changes to RNA secondary structure (Sakumi et al., 2010; Thomas et al., 1998). ITP may also function as a competitive agonist or antagonist for reactions using other nucleotides.

The enzymes belonging to the Maf/Ham1 superfamily hydrolyse ITP to form IMP and pyrophosphate. ITPases hydrolyse ITP, dITP, and the noncanonical nucleotide XTP. IMP can then be used as a substrate for ATP or GTP biosynthesis. Structurally characterized ITPases belonging to the Maf/Ham1 superfamily exist as homodimers and possess a β‐sheet backbone from which two α‐helix lobes extend, forming a substrate‐binding cleft (Stenmark et al., 2007). Highly conserved residues within the substrate‐binding pocket determine substrate specificity. In a comprehensive mutation analysis of human ITPA, Gall et al. (2013) identified key residues that influence substrate specificity.

ITPases have been biochemically and structurally characterized in *E. coli* (rdgB), human (ITPA), and yeast (Ham1). The yeast ITPase is named for its mutant phenotype that shows sensitivity to the mutagenic purine analog, 6‐*N*‐hydroxylaminopurine (HAP). This mutant phenotype was also seen in ITPA knockdown HeLa cells and *E. coli* (Burgis et al., 2003; Menezes et al., 2012). In yeast, HAP sensitivity requires, in addition to mutation of *Ham1*, mutation of a gene necessary for IMP to AMP conversion, *ade12* (Pang et al., 2012). Similarly, in *E. coli* sensitivity to HAP required double mutation of both *rdgB* and *moa*, a gene involved HAP detoxification. Phenotypes of mammalian ITPase mutants can be severe, with ITPase‐deficient mice showing growth retardation and premature death (Sakumi et al., 2010). In humans, many drug interactions can be affected by ITPA deficiency, particularly in the use of purine analogs and the antiviral nucleotide triphosphate ribavirin (Nyström et al., 2018). ITP concentration has been shown to increase in humans under disease conditions following deamination of ATP. Under these conditions, ITP can reach higher levels than adenosine due to a much longer half‐life (Welihinda et al., 2016). ITPA deficiency has also been associated with infantile encephalopathy and increased susceptibility to tuberculosis (Burgis, 2016; Kaur et al., 2019). It is clear that ITPase has a very important role in the cellular maintenance of pools of noncanonical nucleotides.

## 4 WHAT ABOUT THE PLANT HAM1?

To gain a greater understanding of the potential role of plant viral ITPase, it is important to reflect on the role of plant‐encoded ITPases. Unfortunately, no studies of plant endogenous ITPase have been completed. As ITPase is highly conserved across all kingdoms of life, it is likely that ITPase in plants shares a similarly important role in the removal of potentially mutagenic noncanonical nucleotides. However, despite extensive studies in animal systems, there have been no studies on what other important roles plant ITPase may have, particularly in plant disease. In silico data sets show ITPase has nearly ubiquitous expression in *Arabidopsis thaliana*, with highest expression at the vegetative stage and in germinating seeds (Nakabayashi et al., 2005; Schmid et al., 2005; Waese et al., 2017). This correlates well with expression profiles in animals that have shown expression of ITPase in all tissues tested (Behmanesh et al., 2005; Lin et al., 2001).

**FIGURE 2:**
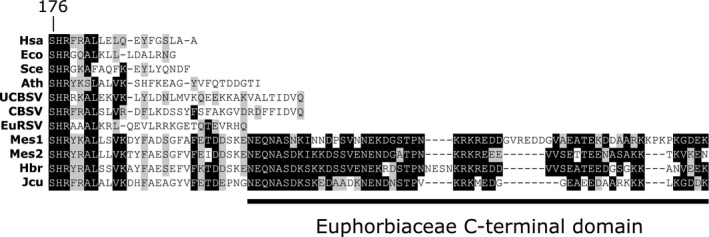
Euphorbiaceae ITPase proteins have a conserved, extended C‐terminal domain that is not present in any other ITPase sequence. The alignment was trimmed at the highly conserved SHR substrate specificity motif. The first residue is labelled for the *Homo sapiens* ITPA sequence. Hsa, *Homo sapiens*, AAK21848.1; Eco, *Escherichia coli*, WP_127472244.1; Sce, *Saccharomyces cerevisiae*, AJR66682.1; Ath, *Arabidopsis thaliana*, NP_001328955.1; UCBSV, ADA61013.1; CBSV, ADR73018.1; Mes, *Manihot esculenta* (cassava), XP_021594792.1, XP_021606115.1; Jcu, *Jatropha curcas*, XP_012077670.1; Hbr, *Hevea brasiliensis*, XP_021644689.1

Cassava encodes two ITPase‐like sequences, one of which shows elevated expression in the shoot apical meristem of healthy plants and the other shows similar expression across all tissues (Wilson et al., 2017). They both share high sequence similarity with characterized plant ITPase sequences with the exception of an additional extended C‐terminal domain that is also found in ITPases encoded by other members of the Euphorbiaceae family, but not in any other ITPases (Figure 2). Potentially, this additional domain is responsible for localization, or in protein–protein interactions. The extended C‐terminus contains a KRKR motif, which is a potential nuclear or membrane localization signal (Do et al., 2006; Kuo et al., 2018).

Several large‐scale expression studies have been completed in cassava in response to infection with cassava brown streak disease (CBSD)‐causal viruses. These studies showed no change in expression of either ITPase‐like gene in response to UCBSV (Amuge et al., 2017) or mixed infection (Anjanappa et al., 2018) and a small increase in expression of a single ITPase gene (cassava4.1_014072m) of approximately 2‐fold in a resistant cassava variety following infection with CBSV (Maruthi et al., 2014). From these results, it does not appear that endogenous Ham1 is regulated by infection with (U)CBSV.

## 5 WHAT DO CBSV, UCBSV, AND EURSV HAVE IN COMMON?

CBSV and UCBSV cause CBSD, which is responsible for devastating losses to cassava crops in Africa. The disease was first described in 1930, originating in Tanzania, and the two causal viruses have since been well characterized, allowing for rapid detection by molecular methods (Monger et al., 2001a, 2001b; Storey, 1936; Tomlinson et al., 2018). EuRSV is a more recent discovery, first reported in the ornamental plant *Euphorbia milii* × *lophogona* in 1976 as causing chlorotic spots or mosaic, leaf, and flower deformations, and reduced growth (Bode & Lesemann, 1976; Marys & Romano, 2011).

The two CBSD causal viruses and EuRSV all have positive‐sense, single‐stranded RNA genomes and share a similar overall genomic structure, as well as all infecting plants within the Euphorbiaceae. All three are transmitted by clonal propagation, grafting or an insect vector, (U)CBSV by cassava whiteflies and EuRSV by aphids. Despite the similarities between the viruses, the genes encoding ITPase are not considered to be homologs. Although it is likely that the genes encoding ITPase in CBSV and UCBSV are evolutionarily related due to their close phylogenetic relationship, the EuRSV Ham1 is distinct. The latter, when coupled with the absence of Ham1 in other members of the *Potyviridae*, suggests that ITPase was acquired independently in these two groups of viruses. It is also possible that CBSV, UCBSV, and EuRSV Ham1 sequences were acquired from each other during coinfection if they shared a common host. Other than in cassava, CBSV and UCBSV have only been detected in a wild cassava relative, but have not been shown to share a common host with EuRSV (Amisse et al., 2019; Knierim et al., 2017).

It is possible that the viruses acquired the ITPase gene from a host plant by horizontal gene transfer (HGT). HGT between host and virus is predicted to have protective functions through either production of a viral antigen by the host or mimicry of a component of the host immune signalling pathways by the virus (Chen et al., 2016). Many examples of HGT of nucleotide metabolic genes from host to virus have been described, including the nucleotide pyrophosphatase, dUTPase (Baldo & McClure, 1999; Wu & Zhang, 2011). Similar to ITP, dUTP is mutagenic if incorporated into DNA and must be metabolized. Viral dUTPases have been shown to function in viral replication to prevent misincorporation of dUTP in cells that have low endogenous dUTPase expression (Hizi & Herzig, 2015).

## 6 IMPORTANCE OF VIRAL HAM1 IN CBSV SYMPTOMOLOGY

Removal of Ham1 from the CBSV genome or mutation of a conserved substrate‐binding motif resulted in striking changes in symptom development in the indicator plant *N. benthamiana*, including a lack of necrosis normally seen in wild‐type infection and leaf curling and chlorotic mottling normally absent from wild‐type infected plants (Tomlinson et al., 2019). Similarly, replacing the CBSV ITPase with the UCBSV ITPase‐encoding sequence also resulted in the same change in phenotype. Virus‐induced necrosis is associated with pathways involved in the hypersensitive response (Komatsu et al., 2010; Mandadi & Scholthof, 2013), suggesting viral ITPase activity may be triggering these signalling pathways. Intriguingly, plants infected with wild‐type UCBSV virus showed a similar phenotype to plants infected with CBSV Ham1 mutant viruses, suggesting the two viruses encode ITPases with different roles in infection; however, no mutant UCBSV virus was tested so any attenuation of symptoms is unknown (Tomlinson et al., 2019). The UCBSV and CBSV Ham1 sequences show low homology, which is probably responsible for the observed differences in symptomatologies (Tomlinson et al., 2018). Research efforts need to focus on the specific sequence differences between the CBSV and UCBSV ITPases that result in such striking variations in symptom development, which currently remain unknown. The changes to symptom development have also yet to be shown in the native host, cassava, which shows drastically different symptomology compared to the indicator host, *N. benthamiana*.

## 7 POSSIBLE ROLES FOR VIRAL HAM1

### 7.1 Theory 1: Reduction of mutation load

RNA viruses have very high mutation rates due to their high rate of replication and the lack of proofreading ability in their encoded RNA polymerase. Within an infected tissue, RNA viruses will exist as a population or viral swarm. The fittest viruses, or fastest replicating viruses, will be most abundant, and the high rate of replication acts as a buffer against potential deleterious mutations, whilst the constraints imposed by cell‐to‐cell movement and transmission may act as selective sweeps. There is no immediately obvious benefit to expressing a house‐cleaning enzyme to prevent a potential increase in mutation rate in RNA viruses. The study by Tomlinson et al. (2019), investigating the effect of the heterologous expression of CBSV ITPase in *N. tabacum* on viral mutation rate, failed to identify any significant difference. Possibly, incorporation of the noncanonical nucleotide into the RNA genome has a greater effect on RNA structure and thus the rate of translation (Sakumi et al., 2010; Thomas et al., 1998). The study by Tomlinson et al. (2019) did find a delayed increase in transcript abundance for mutant viruses lacking ITPase compared to wild‐type viruses, which could potentially be a result of reduced translation efficiency of viral proteins involved in viral RNA replication.

While enzymes involved in maintaining RNA replication fidelity are rare, coronaviruses encode an enzyme with 3′→5′ exoribonuclease activity, probably involved in proofreading. Intriguingly, reduced activity of this enzyme sensitizes the virus to mutation through treatment with the synthetic mutagen 5‐fluorouracil and the purine analog ribavirin, whose harmful effects are both known to be attenuated by ITPase activity (Carlsson et al., 2013; Hitomi et al., 2011; Smith et al., 2015). This potentially suggests that the proofreading ability reduces the influence of ITP on RNA replication fidelity. It is also important to note that not all viral RNA‐dependent RNA polymerases (RdRps) show similar levels of fidelity (Smith, 2017). However, (U)CBSV RdRp fidelity has not been directly compared with RdRps from other ipomoviruses lacking Ham1. Possibly, the acquisition of ITPase is a result of a low‐fidelity RdRp.

A possible reason for the lack of increase in mutation frequency could be that Tomlinson et al. (2019) tested this in a nonhost plant (tobacco) and under laboratory conditions. Cassava can naturally grow successfully under environmentally challenging (high temperatures and drought) conditions and this could result in an increased accumulation of noncanonical nucleotides through oxidative deamination. There have been no studies of changes to dITP/ITP pools following exposure to stress in plants. It is possible that endogenous ITPase expression in Eurphobiaceae is lower compared to that in other plants, resulting in increased accumulation of ITP, which could result in a higher mutation frequency to the infecting viral genome. If the extended C‐terminal domain is in fact a nuclear localization signal (Figure 2), this would suggest that Euphorbiaceae ITPases are specifically influencing the dITP pools in the nucleus to prevent incorporation into DNA. Cytosolic pools of ITP would not be reduced by the endogenous ITPase and could be incorporated in the viral genome. This could explain why ITPase is not found in other viral genomes and is restricted to viruses infecting members of the Euphorbiaceae family.

### 7.2 Theory 2: Disruption of plant signalling pathways

Several studies have shown that cassava responds to (U)CBSV infection with increased expression of genes belonging to the pathogenesis‐related (PR) gene family (Amuge et al., 2017; Anjanappa et al., 2018; Irigoyen et al., 2020). PR proteins are frequently used as biomarkers for defence signalling pathways and are associated with increases in plant hormones such as salicylic acid (SA) (Ali et al., 2018). Furthermore, phytohormone genes are over‐represented in the transcriptional response of cassava to CBSV, also suggesting involvement of signalling pathways following infection (Maruthi et al., 2014). It is clear that signalling pathways are altered following infection with (U)CBSVs; however, what is triggering these changes is unknown. Intriguingly, CBSD causal viruses lack HC‐Pro, a gene present in many other *Potyviridae* genomes that has been shown to be involved in suppression of SA‐mediated defence responses (Murphy et al., 2020; Poque et al., 2018) (Figure 1).

Cyclic GMP (cGMP) has been shown to increase expression of PR genes in *N. tabacum* and also promotes ethylene production and perception of ethylene. While ITP has not been shown to influence cGMP synthesis, ITP has been shown to result in increased levels of cAMP through agonistic engagement with adenosine receptors and stimulation of adenylate cyclase (Welihinda et al., 2016). Few adenylate cyclases have been characterized in plants because many exist within multifunctional, multidomain proteins and are therefore difficult to identify by homology (Bianchet et al., 2019), so the role of cAMP in plants has not been extensively studied. Knockout of cyclic nucleotide‐gated channels results in increased sensitivity to biotic and abiotic stresses, including reduced resistance to plant viruses and, in *A. thaliana*, stress‐responsive proteins show differential expression in response to treatment with cAMP (Alqurashi et al., 2016; Gehring, 2010; Saand et al., 2015; Thomas et al., 2013). cAMP and cGMP have been shown to regulate the pathogen‐responsive phenylpropanoid pathway and cassava infected with CBSV shows changes to genes involved in phenylpropanoid biosynthesis (Maruthi et al., 2014; Pietrowska‐Borek & Nuc, 2013). It is possible that ITPase indirectly prevents the accumulation of cAMP and blocks downstream signalling pathways because IMP is a poor agonist compared to ITP in interactions with adenosine receptors and, therefore, is unlikely to influence cAMP levels. In addition to influencing cAMP synthesis, ITP has been shown to function in the activation of G‐proteins and could act a signal amplifier in plant response to infection (Klinker & Seifert, 1997). G‐proteins are known to play a role in plant defence against fungal, bacterial, and viral pathogens by regulating phytohormone production and can also influence cAMP synthesis (Brenya et al., 2016).

### 7.3 Theory 3: Reduction of ITP antagonistic/agonistic interactions with nucleotide‐binding proteins

In addition to the potential role of ITP in G‐protein and cyclic nucleotide signalling, ITP may function as an antagonist/agonist for other GTP‐ or ATP‐binding proteins. Nucleotide‐binding proteins have enormously diverse functions in cellular processes, in addition to roles in signalling (Xiao & Wang, 2016). For example, the *E. coli* ITPase, YjjX, has been shown to interact with the GTPase, elongation factor Tu (EF Tu), which is involved in peptide elongation (Zheng et al., 2005). *E. coli* ITPase is predicted to interact with EF Tu via bound GTP to prevent formation of the EF‐TuGTP‐aminoacyl‐tRNA complex and prevent peptide elongation. This is predicted to be a protective role in prevention of synthesis of misfolded proteins, similarly to when cells undergo oxidative stress (Sanchez et al., 2019). Viral polyprotein biosynthesis requires the interaction of viral genome‐linked protein (VPg) and the host’s eukaryotic translation initiation factor 4E (eIF4E) isoforms. Mutations in cassava eIF4E result in attenuated CBSV virulence (Gomez et al., 2019). Possibly, viral ITPase is interacting with a GTP‐dependent enzyme involved in translation similarly to *E. coli* ITPase, such as EF Tu, Eukaryotic Initiation Factor 2, or the mRNA capping enzyme.

ITP can also function in the assembly of microtubules (Muraoka et al., 1999; Muraoka & Sakai, 1999). Changes to microtubule dynamics following viral infection have been demonstrated to be a general phenomenon, occurring in animals, bacteria, and plants (Naghavi & Walsh, 2017). Microtubule dynamics in plant viral infection have been shown to impact viral movement (Boyko et al., 2000), vector transmission (Blanc et al., 1996), and viral replication (Mas & Beachy, 1999). Microtubule polymerization is a dynamic process involving end‐to‐end addition of α‐ and β‐tubulin subunits. Assembly requires binding and hydrolysis of GTP, but ITP is a functional substitute (Muraoka et al., 1999; Muraoka & Sakai, 1999). While the affinity for GTP is much higher than for ITP, the critical concentration is much lower for ITP (Chakrabarti et al., 2000). Viral ITPase activity could potentially be involved in regulation of microtubule polymerization via reduction of the pool of ITP, which is possibly increased in response to infection. Furthermore, a gene expression analysis study of UCBSV‐infected cassava showed changes in expression to microtubulin biosynthetic genes (Amuge et al., 2017). Changes to microtubulin biosynthetic genes were observed in the resistant line and no changes were shown in the sensitive line, suggesting that microtubule dynamics may be important for the plant response to viral infection (Amuge et al., 2017).

## 8 CONCLUSIONS AND FUTURE WORK

ITPases are highly conserved across all kingdoms of life and are important enzymes for prevention of mutagenic effects caused by accumulation of noncanonical nucleotides. ITPases have recently been discovered in three plant RNA viruses, but their biological role remains a mystery (Knierim et al., 2017; Mbanzibwa et al., 2009). As RNA viruses encode very few genes and will not maintain unnecessary genetic information within their genomes, is highly likely that these viral‐encoded ITPases carry out an important function. Evidence from analysis of mutant viruses shows changes to the disease phenotype, suggesting that ITPases modulate the plant response to the virus (Tomlinson et al., 2019). Understanding their function may assist in the development of virus‐resistant cassava, an essential quest to improve food security across sub‐Saharan Africa (Tomlinson et al., 2018).

Here we have proposed three theories for the potential function of ITPase during infection by the Euphorbiaceae‐specific viruses (U)CBSV and EuRSV: prevention of mutagenic effects of an increased pool of ITP, disruption of plant signalling following infection, and disruption of other potential cellular roles of ITP. Further research should target these as well as the function and expression profiles of plant‐encoded ITPase, particularly in the host plants for (U)CBSV and EuRSV. It is unclear if viral ITPase expression is important as a result of reduced expression of endogenous ITPase, potentially resulting in increased pools of cellular ITP. Viral sequences do not appear to show increased levels of mutation; however, this has yet to be shown in the host plant. Potentially, these viruses, which infect members of the Euphorbiaceae family, have acquired ITPase in response to a host‐specific regulation of ITP pools. A greater understanding of the host's maintenance of cellular ITP will help to elucidate a role for viral Ham1.

## Data Availability

Data sharing is not applicable to this article as no new data were created or analysed in this study.
